# Measuring dopaminergic function in the 6-OHDA-lesioned rat: a comparison of PET and microdialysis

**DOI:** 10.1186/2191-219X-3-69

**Published:** 2013-10-02

**Authors:** Matthew D Walker, Katherine Dinelle, Rick Kornelsen, Anna Lee, Matthew J Farrer, A Jon Stoessl, Vesna Sossi

**Affiliations:** 1 Department of Physics and Astronomy, University of British Columbia, 6224 Agricultural Road, Vancouver, British Columbia V6T 1Z1, Canada; 2 Pacific Parkinson's Research Centre, University of British Columbia, 2221 Wesbrook Mall, Vancouver, British Columbia V6T 2B5, Canada; 3 Department of Medical Genetics, University of British Columbia, Vancouver, British Columbia V6H 3N1, Canada

**Keywords:** Dopamine synthesis, FDOPA, Parkinson's disease, PET, Microdialysis, 6-OHDA lesion

## Abstract

**Background:**

[^18^ F]fluorodopa (FDOPA) positron emission tomography (PET) allows assessment of levodopa (LDOPA) metabolism and is widely used to study Parkinson's disease. We examined how [^18^ F]FDOPA PET-derived kinetic parameters relate the dopamine (DA) and DA metabolite content of extracellular fluid measured by microdialysis to aid in the interpretation of data from both techniques.

**Methods:**

[^18^ F]FDOPA PET imaging and microdialysis measurements were performed in unilaterally 6-hydroxydopamine-lesioned rats (*n* = 8) and normal control rats (*n* = 3). Microdialysis testing included baseline measurements and measurements following acute administration of LDOPA. PET imaging was also performed using [^11^C]dihydrotetrabenazine (DTBZ), which is a ligand for the vesicular monoamine transporter marker and allowed assessment of denervation severity.

**Results:**

The different methods provided highly correlated data. Lesioned rats had reduced DA metabolite concentrations ipsilateral to the lesion (*p* < 0.05 compared to controls), with the concentration being correlated with FDOPA's effective distribution volume ratio (EDVR; *r* = 0.86, *p* < 0.01) and DTBZ's binding potential (BP_ND_; *r* = 0.89, *p* < 0.01). The DA metabolite concentration in the contralateral striatum of severely (>80%) lesioned rats was lower (*p* < 0.05) than that of less severely lesioned rats (<80%) and was correlated with the ipsilateral PET measures (*r* = 0.89, *p* < 0.01 for BP_ND_) but not with the contralateral PET measures. EDVR and BP_ND_ in the contralateral striatum were not different from controls and were not correlated with the denervation severity.

**Conclusions:**

The demonstrated strong correlations between the PET and microdialysis measures can aid in the interpretation of [^18^ F]FDOPA-derived kinetic parameters and help compare results from different studies. The contralateral striatum was affected by the lesioning and so cannot always serve as an unaffected control.

## Background

For three decades, [^18^ F]fluoro-3,4-dihydroxyphenyl-l-alanine ([^18^ F]FDOPA) positron emission tomography (PET) has served as an imaging biomarker for dopamine (DA) synthesis, storage, and turnover in the human brain [1-6]. More recently, [^18^ F]FDOPA PET has been used in the study of mouse [7,8] and rat [9,10] models of Parkinson's disease (PD). The increasing availability of transgenic rodent models of PD which may recapitulate the cardinal features of the disease, including disease progression, has spurred new interest in imaging the dopaminergic system of rodents. Non-invasive molecular imaging such as PET enables longitudinal measurements in rodent models to be made and facilitates a direct comparison with the human disease. [^18^ F]FDOPA traces the metabolic pathway of exogenous levodopa (LDOPA), providing information that is quite distinct from that given by ligands which bind to either dopamine receptors, transporters, or other targets within the dopaminergic system. One such ligand is (+)-dihydrotetrabenazine (DTBZ), which binds to the vesicular monoamine transporter 2 (VMAT2). [^11^C]DTBZ binding informs on the density of VMAT2, from which DA terminal integrity (i.e., denervation severity) may be inferred [11,12]. Insights into the function of the dopaminergic system can also be made by microdialysis. The insertion of dialysis probes directly into the rodent brain provides a means of sampling extracellular fluid (ECF) which can then be assayed for various compounds, including its dopamine and dopamine metabolite content. The direct sampling of ECF and the quantification of DA and DA metabolite concentrations within such samples is a commonly available and well-used technique for *in vivo* research, but the method is invasive and not well suited to longitudinal investigations [13-15].

The results from microdialysis are relatively simple to interpret as compared to the outputs from the kinetic modeling of dynamic [^18^ F]FDOPA PET data, in which a compartmental model is typically used to describe several key steps that govern the tracer's time-course in the brain. These steps include, among others, (1) transport of the tracer across the blood–brain barrier as mediated by the large neutral amino acid transporter [16]; (2) decarboxylation of FDOPA to DA, catalyzed by aromatic amino acid decarboxylase (AADC); (3) incorporation and storage of DA into vesicles [17], mediated by the vesicular monoamine transporter (type 2) [18]; (4) vesicular release of DA into the synapse and its subsequent metabolism via catechol-*O*-methyltransferase (COMT) and monoamine oxidase (MAO) [19] either within the synapse or after reuptake by the dopamine transporter [20]; and (5) washout of metabolites into the circulation. Due to the complex pathway traced by [^18^ F]FDOPA, many simplifications are made to allow robust parameter estimates to be obtained from the kinetic model. These simplifications necessarily lead to greater ambiguity in the biological interpretations for the various parameters. Given these complexities, a direct comparison between [^18^ F]FDOPA PET and *in vivo* microdialysis in the same animals is of great value.

In the current investigation, we make a comparison between results from [^18^ F]FDOPA PET, [^11^C]DTBZ PET, and *in vivo* striatal microdialysis to directly measure DA and DA metabolites in normal control rats and in rats unilaterally 6-hydroxydopamine (6-OHDA)-lesioned at the substantia nigra pars compacta (SNc) [21,22]. We aim to allow microdialysis data, either from previous literature or future studies, to be put into perspective alongside results from [^18^ F]FDOPA and [^11^C]DTBZ PET.

## Methods

### Animals, 6-OHDA lesioning, and microdialysis surgery

Procedures involving animals were approved by the University of British Columbia's ethics committee and followed the guidelines of the Canadian Council on Animal Care. We report data from three normal control (age-matched, not sham-operated) and eight unilaterally 6-OHDA-lesioned [21,22] rats (Sprague Dawley, males, from Charles River, St. Constant, Quebec, Canada). These rats underwent multiple PET imaging sessions, as reported in Walker et al. [10], before undergoing two microdialysis sessions. The animals had free access to standard diet and tap water. They were housed at 21°C with a 12-h light cycle (light from 7 a.m. to 7 p.m.). Dopaminergic denervation was produced by injection of 10 μg of 6-OHDA hydrobromide (Sigma-Aldrich, St. Louis, MO, USA) dissolved in 4 μL of 0.05% ascorbic acid solution. The injection co-ordinates (SNc) were as follows: anteroposterior (AP) −4.7 mm (from the bregma), mediolateral (ML) −1.5 mm (from the midline), and dorsoventral (DV) −7.9 mm (from the skull surface) [23]. To protect noradrenergic nerve terminals, desipramine (Sigma-Aldrich) was given at least 30 min prior to surgery (25 mg/kg intraperitoneally (i.p.)). This ensured selectivity for dopaminergic neurons [24]. Rats were 2.4 ± 0.5 months (average and standard deviation) old at the time of lesioning. Denervation severity was determined by [^11^C]DTBZ PET imaging, which was performed at least 1 month after lesioning (2.1 ± 0.8 months) at which time 6-OHDA-induced denervation is considered relatively stable. The [^18^ F]FDOPA PET imaging sessions were also performed at least 1 month after lesioning (average of 3.5 months, maximum of 8.5 months post lesioning). Microdialysis was performed after completion of all PET imaging at 12 ± 3.6 months post lesioning. Surgery was performed for implantation of a microdialysis guide cannula (CMA 7, Harvard Apparatus, Holliston, MA, USA) into the left and right striata at co-ordinates AP +1.0 from the bregma, ML ±2.6 from the bregma, and DV −3.0 from the skull. A headcap was built up around the cannula using dental cement (Jet Acrylic, Lang Dental, Wheeling, IL, USA) and secured to the skull with five self-tapping bone screws (shaft diameter 1.17 mm, shaft length 4.7 mm, Fine Science Tools, North Vancouver, Canada). All surgeries were performed under isoflurane anesthesia. Ketoprofen (5 mg/kg sc.) was given for pain relief, and a local analgesic (bupivacaine, 2.5 mg/kg) was applied at the incision site.

### Microdialysis

Following headcap surgery, rats were allowed 36 h of recovery. Microdialysis was then performed under isoflurane anesthesia twice in each rat within the next 4 days, with at least 1 day of recovery between microdialysis sessions. The use of anesthesia replicated the conditions during PET scanning and simplified the microdialysis procedure. Each session began with the insertion of a microdialysis probe (CMA 7, Harvard Apparatus) into each guide cannula. These probes have a shaft length of 7 mm with a 2-mm-long cuprophane membrane (cutoff of 6,000 Da) of 0.24-mm outer diameter. Probes had been previously prepared by flowing sterile artificial cerebrospinal fluid (aCSF) through them (CMA, Harvard Apparatus). The aCSF flow rate was 0.5 μL/min, but 0.9 μL/min in a few rats where extra dialysate was required (for other testing not reported here). Dialysate collection began 2 h after insertion of the probe to allow some recovery from potential tissue damage caused by probe insertion. In both sessions, 1 h of baseline data was first collected. This was followed by injection (i.p.) of 40 mg/kg entacapone (which inhibits peripheral COMT) in one session and of benserazide/LDOPA (15 and 50 mg/kg, respectively, with the LDOPA given 30 min after the peripheral AADC inhibitor) in the other session. Entacapone was obtained from a local pharmacy in pill form and ground to a fine powder. Benserazide (Sigma-Aldrich), LDOPA (Sigma-Aldrich), and entacapone were mixed in distilled water, 0.05% ascorbic acid, and saline, respectively, for injected volumes of 1 mL/kg.

Dialysates were collected from each probe at intervals of 24 min and immediately injected (9 μL) onto a high-performance liquid chromatography system consisting of a pump (Series IV, Rose Scientific, Edmonton, Canada), pulse damper (Scientific Systems Inc., State College, PA, USA), injector with 20-μL injection loop (Rheodyne, IDEX Health & Science, Oak Harbor, WA, USA), reverse-phase column (TSKgel super-ODS, 2-μm silica particles, 110-Å pores, 2-mm i.d. × 10-cm length, Tosoh Bioscience, San Francisco, CA, USA), and electrochemical detector (Decade II, Antec, Fremont, CA, USA). The flow rate was 0.18 mL/min, with a pressure of approximately 1,600 psi. Each liter of mobile phase contained 100 mL methanol, 900 mL water, 6 g sodium acetate, 40 mg ethylenediaminetetraacetic acid (EDTA), 4 mg sodium dodecyl sulfate (SDS), and glacial acetic acid (to pH 4.0). The electrochemical cell (VT-03, Antec) had a 2-mm glassy carbon working electrode with a salt bridge reference electrode. A 25-μm spacer was used. The applied voltage was +650 mV; the cell and column were maintained at 38°C. Data were collected and analyzed using EZChrom Elite (Agilent, Santa Clara, CA, USA).

Typical probe recoveries as measured at room temperature *in vitro* were 24%, 26%, and 24% for DA and the DA metabolites 3,4-dihydroxyphenylacetic acid (DOPAC) and homovanillic acid (HVA), respectively, at 0.5-μL/min flow rate. The recoveries were, on average, 14% at 0.9 μL/min. The *in vitro* measured probe recoveries were used as estimates of the *in vivo* probe recoveries, thus correcting for differences in recovery between probes and allowing the relative dialysate concentrations to be compared across rats. The procedure does not provide a highly accurate measurement of the absolute concentration in the ECF since the *in vivo* recoveries are known to differ from those measured *in vitro*. Recoveries were combined with the measured calibration factor for each compound to produce the concentrations reported here. The data include those from baseline (average of both sessions), the percentage change from baseline following injection of benserazide/LDOPA (one session), and the percentage change from baseline following injection of entacapone (one session). The post-drug concentrations were found by averaging data from 100 to 180 min post benserazide injection (70 to 150 min post LDOPA injection) and from 90 to 150 min post entacapone injection. Reported times are corrected for the time required for the sample to travel from the probe to the collection vial. In our microdialysis experiments using LDOPA, we did not pre-dose with a COMT inhibitor since the chromatographic analysis can separate DA and DA metabolites from 3-*O*-methyldopa and because a relatively high dose of LDOPA was used. The testing allowed assessment of the basal concentrations, the central effects (if any) of entacapone, and the increase in DA metabolite concentrations following LDOPA.

### PET scanning and image analysis

Extensive details of the PET imaging and analysis procedures are reported in Walker et al. [10] and are summarized here. The results reported here are from one [^11^C]DTBZ scan and two [^18^ F]FDOPA PET scans. For the [^18^ F]FDOPA scans, a COMT inhibitor (40 mg/kg entacapone) and an AADC inhibitor (10 mg/kg benserazide) were given i.p. prior to the administration of the radiotracer (at 90 and 30 min, respectively). The scans are a subset of those reported previously in Walker et al. [10], namely the scans from rats that were subsequently available for microdialysis and which did not include administration of tolcapone prior to [^18^ F]FDOPA PET. Scanning was conducted under 2.5% isoflurane gas anesthesia using the MicroPET Focus120 small animal scanner (Concorde/Siemens, Knoxville, TN, USA) [25]. Emission data were collected for 3 h ([^18^ F]FDOPA) or 1 h ([^11^C]DTBZ) and split into 26 or 17 frames, respectively. Frames had increasing durations, in the range 30 to 900 s ([^18^ F]FDOPA) and 30 to 480 s ([^11^C]DTBZ). Data were fully corrected for randoms, attenuation, scatter, normalization, and dead time. Images were reconstructed by filtered backprojection. The spatial resolution within the resulting images is <1.5-mm full width at half maximum [25]. Blood was sampled from the tail during [^18^ F]FDOPA scanning and assayed for radiolabeled metabolites [26], following which a small fraction of scans were discarded due to high concentrations of the radiolabeled metabolite 3-*O*-methyl-6-[^18^ F]fluoro-l-dopa ([^18^ F]OMFD; >25%) or low concentrations of the parent compound (<40%) at 160 min post tracer injection (i.e., in scans for which peripheral COMT and/or AADC inhibition was insufficient). Despite this, all rats had at least one [^18^ F]FDOPA scan that was retained (most had two, in which case the average result is reported).

PET images were analyzed using the ASIPro software (CTI Concorde Microsystems). For each scan, three regions of interest (ROIs) were placed on summation radioactivity images, defining the left and right striata and the cerebellum. The ROI volumes were 0.022 cm^3^ (striatal) and 0.043 cm^3^ (cerebellum). In contrast to the data reported in [10], the ROI for the cerebellum used for PET data analysis was placed with the guidance of a co-registered atlas [27]; this improved the consistency of the data. Kinetic modeling and analysis was performed using Matlab (The Mathworks, Natick, MA, USA) with in-house software.

[^11^C]DTBZ was analyzed using Logan graphical analysis with a cerebellum reference region [28], which estimates the distribution volume ratio (DVR). The time after which data are fitted, t*, was set to 30 min, and the ${\bar{k}}_{2}$ term was omitted [29]. The binding potential of DTBZ (BP_ND_) was calculated as BP_ND_ = DVR – 1.

[^18^ F]FDOPA was similarly analyzed using the Logan plot, with the modification that the time-activity curve (TAC) from the reference region was subtracted from the striatal TAC prior to running the analysis [6]. The estimated slope equals the effective distribution volume ratio (EDVR), which informs on the distribution volume of the striatal 6-[^18^ F]fluorodopamine (FDA) compartment as compared to the reference region.

Extended Patlak graphical analysis [5,30], which includes a term describing the loss of radiolabeled metabolites from the sequestered compartment, was also applied to the [^18^ F]FDOPA data. The analysis provided estimates of *k*_ref_, the rate constant that models the decarboxylation of FDOPA to FDA and the sequestration of FDA in vesicles.

The asymmetry for results from both PET and microdialysis was calculated as 1 minus the ratio of the value from the ipsilateral side to the contralateral side; normal control rats were hence expected to have an asymmetry of 0 for all measures, with severely lesioned rats likely to have asymmetries closer to 1 (maximum asymmetry). The asymmetry in the DTBZ PET measure provides the assessment of denervation severity [11,12].

### Correlation analysis and statistical testing

Data from PET and microdialysis were subject to regression analysis using Matlab (The MathWorks). In each case, Pearson's product–moment correlation coefficient (*r*) was calculated, along with the probability of obtaining the measured correlation by chance if the true correlation was 0. Group comparisons between rats were also made, with group assignment for lesioned rats based on the DTBZ PET denervation severity measure. For these inter-group comparisons (three groups, *n* ≥ 3 in each group), statistical analysis followed Fisher's method of analysis of variance (ANOVA) followed by *post hoc* comparisons using the least significant difference technique when the omnibus test found a significant difference [31].

## Results

For microdialysis measures, the concentration of DOPAC was highly correlated with the concentration of HVA (*r* = 0.83, *p* < 0.01) across all rats, with a relationship not significantly different from direct proportionality. These two DA metabolites were thus combined for subsequent analysis.

**Figure 1:**
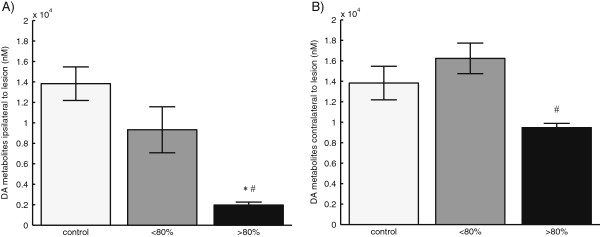
**Basal microdialysis measurements.** Basal DA metabolite levels measured by microdialysis in the striatum, estimated using *in vitro* probe recoveries. **(A)** Results for striatum ipsilateral to the 6-OHDA lesion. **(B)** Results for striatum contralateral to the 6-OHDA lesion. The asterisk indicates significant difference from the unlesioned control group, and the number sign indicates significant difference from the <80% lesion group (*p* < 0.05). For unlesioned control rats, data from the left and right striata were averaged. Error bars represent standard errors on the mean.

Figure 1 shows the impact of 6-OHDA lesioning on DA metabolite concentrations as measured in the ECF by microdialysis. Lesioning caused a decrease in the metabolite concentration ipsilateral to the lesion, with the level for severely lesioned (>80%) rats being significantly lower than those for both the control group and the <80% lesion group. On the contralateral side, the severely lesioned rats had the lowest metabolite concentration across groups, with the difference being significant when compared to the <80% lesion group. Although the DA metabolite concentration measured contralateral to the lesion in the <80% lesion group was higher than the concentration measured in controls, the difference was not significant.

**Figure 2:**
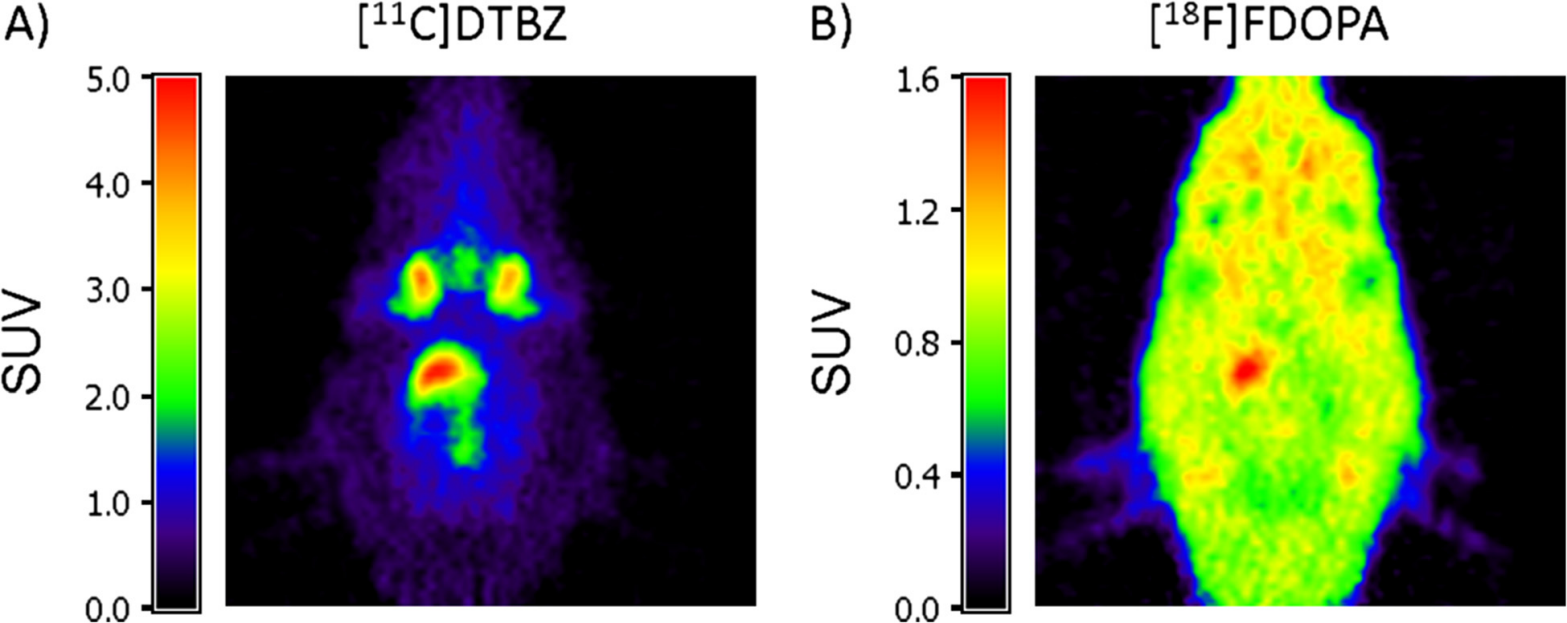
**Example PET images.** PET images of a rat with a severe (90%) lesion. **(A)** [^11^C]DTBZ image. **(B)** [^18^ F]FDOPA image. Both images are shown in units of SUV. The images show a horizontal section through the striatum; the intact (left) striatum is the region with the highest uptake in both images.

Example PET images from a rat with a lesion of severity 0.90 (DTBZ PET measure) are shown in Figure 2. For each tracer, the displayed images are summation images from the entire scan, with the color scale set to the standard uptake value (SUV; equal to the activity concentration divided by the injected dose per unit body weight (kBq/cm^3^ per MBq/kg)).

**Figure 3:**
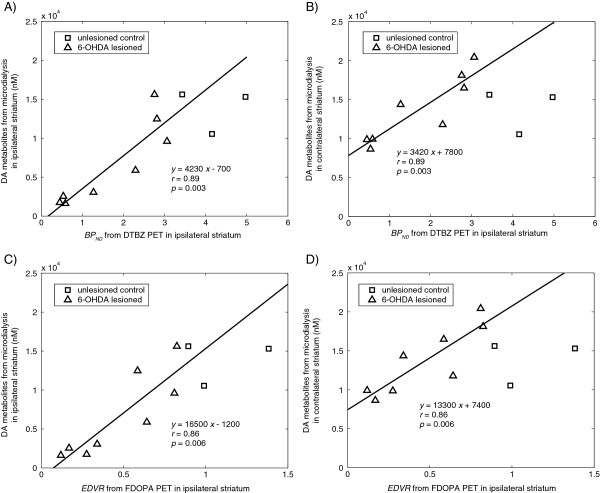
**Correlation between basal microdialysis and PET. (A)** Correlation between basal, ipsilateral DA metabolite concentrations and the DTBZ PET-measured BP_ND_ in the ipsilateral striatum. **(B)** Correlation between basal, contralateral DA metabolite concentrations and the DTBZ PET-measured BP_ND_ in the ipsilateral striatum. **(C)** Correlation between basal, ipsilateral DA metabolite concentrations and the FDOPA PET-measured EDVR in the ipsilateral striatum. **(D)** Correlation between basal, contralateral DA metabolite concentrations and the FDOPA PET-measured EDVR in the ipsilateral striatum. In all four graphs, the solid line represents the regression to the data from 6-OHDA-lesioned rats.

Basal DA metabolite levels measured by microdialysis, both ipsilateral and contralateral to the 6-OHDA lesioning, were strongly correlated with striatal PET measurements ipsilateral to the lesion. Conversely, the PET measures (BP_ND_ from DTBZ PET, EDVR from FDOPA) on the contralateral side appeared not affected by the lesioning, with values not different from those for normal control rats and not correlated with denervation severity nor with the measured DA metabolite levels either ipsilateral or contralateral to the lesion. The correlations to ipsilateral PET measurements for 6-OHDA-lesioned rats are shown in Figure 3, along with the various correlation coefficients and regression lines (all *r* ≥ 0.86, *p* ≤ 0.006). When the FDOPA PET-measured *k*_ref_ was considered rather than the EDVR, the correlation with basal DA metabolite levels was only slightly reduced (*r* = 0.83, *p* = 0.010) as compared to that for the EDVR (*r* = 0.86, *p* = 0.006) in the ipsilateral striatum. There was a trend (not significant) for the ipsilateral *k*_ref_ to be correlated with the contralateral basal DA metabolite concentration (*r* = 0.65, *p* = 0.08). These results demonstrate that for 6-OHDA-lesioned rats, microdialysis-measured DA metabolite concentrations in the striatum contralateral to the lesioning are correlated with PET measures in the ipsilateral striatum. This occurred in the absence of a corresponding decrease in the BP_ND_ from DTBZ or the EDVR from FDOPA PET within the same contralateral striata.

**Figure 4:**
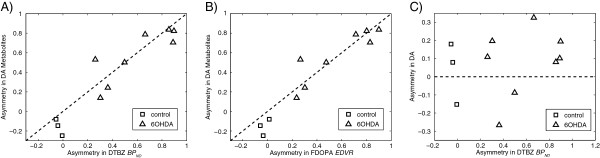
**Asymmetry in microdialysis compared to PET. (A)** Asymmetry in basal DA metabolite levels (DOPAC + HVA) compared to asymmetry in DTBZ BP_ND_. **(B)** Asymmetry in basal DA metabolite levels (DOPAC + HVA) compared to asymmetry in FDOPA EDVR. **(C)** Asymmetry in basal DA vs. asymmetry in DTBZ BP_ND_. Dashed lines in **(A)** and **(B)** show the line of identity, while in **(C)** the dashed line shows asymmetry equal to 0.

The asymmetry in the data was also considered, in part, to reduce noise in the data that could be caused by systematic errors affecting both striata in an individual scan. As shown in Figure 4, there was a significant correlation (*r* = 0.93, *p* < 0.001) between asymmetry in DA metabolites and asymmetry in DTBZ BP_ND_. The line of identity was contained within the bounds of the 95% confidence limit on the line of best fit, despite the aforementioned observations that DA metabolites in the contralateral striatum are correlated with the DTBZ BP_ND_ in the ipsilateral striatum (thus reducing slightly the asymmetry). Similar results (*r* = 0.95, *p* < 0.001) were found when comparing the asymmetry in DA metabolites to the asymmetry in the EDVR from FDOPA. On the other hand, there was no asymmetry observed in DA concentrations as seen in Figure 4C. The mean asymmetry for the DA concentration in lesioned rats was only 8%, not significantly different from 0, with no trend for increased asymmetry with increasing denervation severity. There was a trend for the total DA concentration in ECF (average from both striata) to be reduced with decreasing ipsilateral DTBZ BP_ND_, but this trend was not statistically significant (*r* = 0.53, *p* = 0.09). The average (median) DA concentration in ECF for normal control rats was estimated at 60 nM, and that for lesioned rats was 43 nM. The asymmetry in DTBZ BP_ND_ provided our measure of denervation severity; it ranged from 0.26 to 0.90 in these rats.

The contralateral DTBZ BP_ND_ was not correlated with the ipsilateral DTBZ BP_ND_ (*r* = 0.1), which further confirmed that the unilateral injection of 6-OHDA did not lead to a bilateral lesion; more specifically, it did not cause a bilateral reduction in dopaminergic terminal density. A trend was observed however in FDOPA *k*_ref_ when comparing the two sides of the brain (*r* = 0.47, *p* = 0.12), although there was no such correlation for the EDVR (*r* = 0.1).

**Figure 5:**
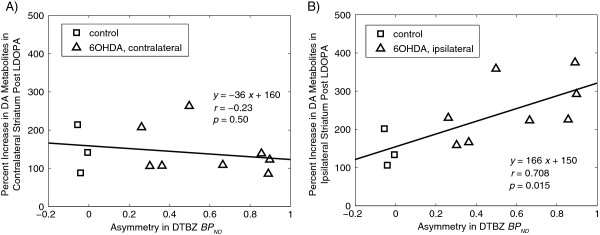
**Microdialysis following acute LDOPA.** Increases in DA metabolite concentrations in striatal dialysates (at time *t* = 120 to 180 min) following i.p. injection of 15 mg/kg benserazide (at *t* = 0 min) and 50 mg/kg LDOPA (at *t* = 30 min), compared to denervation severity (measured as the PET-measured asymmetry in DTBZ binding). **(A)** Results for the left striatum (contralateral to the lesion). **(B)** Results for the right striatum (ipsilateral to the lesion). The solid lines in **(A)** and **(B)** show linear regressions to the data.

Increases in dopamine metabolite concentrations (DOPAC + HVA) following i.p. injection of benserazide/LDOPA were observed by microdialysis. These increases are regressed against the DTBZ PET-measured denervation severity in Figure 5. For the striatum contralateral to the lesion, the increase in DA metabolite concentrations did not depend on the severity of the lesion and averaged 144%. For the ipsilateral striatum, a significant, positive correlation was found between the denervation severity (measured as the asymmetry in DTBZ binding) and the percentage increase in DA metabolite levels (*r* = 0.7, *p* = 0.015). For lesioned rats, the percentage increase in DA metabolites in the ipsilateral striatum was not correlated with the increase in the contralateral striatum (*n* = 8, *r* = 0.32, *p* = 0.4); this is unlike the normal control rats where increases tended to be similar between the left and right striata (as would be expected).

**Figure 6:**
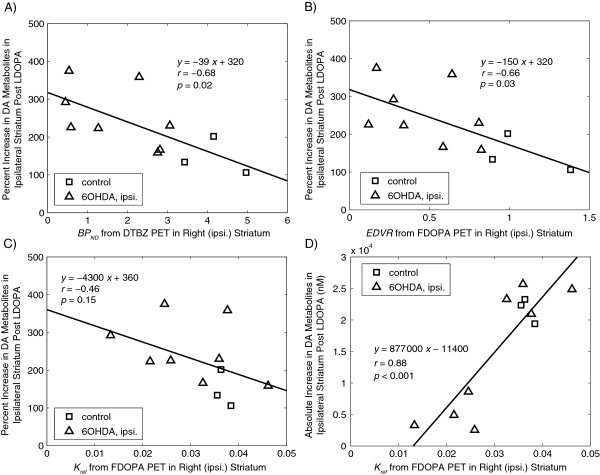
**Microdialysis-measured effects of acute LDOPA compared to functional measures from PET.** Correlations between the increases in DA metabolite concentrations in striatal dialysates and PET measures of dopaminergic function. Data are for the striatum ipsilateral to lesioning, following i.p. injection of 15 mg/kg benserazide (*t* = 0) and 50 mg/kg LDOPA (*t* = 30 min). **(A)** Comparison with DTBZ BP_ND_. **(B)** Comparison with FDOPA EDVR. **(C)** Comparison with FDOPA *k*_ref_. **(D)** Comparison of the absolute increase in DA metabolites with FDOPA *k*_ref_. In each panel, a solid line shows the result of a linear regression to the data.

The percentage increases in the microdialysis-measured DA metabolite concentrations in the ipsilateral striatum are also shown in Figure 6 where they are compared against the estimates of DTBZ BP_ND_ (Figure 6A), FDOPA EDVR (Figure 6B), and FDOPA *k*_ref_ (Figure 6C) as estimated for the ipsilateral striatum by PET. Significant correlations were found between the percentage increase in DA metabolites and the DTBZ BP_ND_ (*r* = −0.68, *p* = 0.02) and FDOPA EDVR (*r* = −0.66, *p* = 0.03), with a non-significant trend observed in the regression against FDOPA *k*_ref_ (*r* = −0.46, *p* = 0.15). Figure 6D shows the absolute increase in DA metabolites (rather than the percentage change) following LDOPA as compared with FDOPA *k*_ref*.*_ A strong correlation was found between these measures (*r* = 0.88, *p* < 0.001). This correlation was slightly stronger than the correlations between the absolute increase in DA metabolite concentrations and the FDOPA PET-measured EDVR (*r* = 0.77, *p* = 0.006) and the DTBZ BP_ND_ (*r* = 0.81, *p* = 0.003).

Our tests with injection of 40 mg/kg entacapone alone found no significant changes in DA and DA metabolite concentrations in ECF as compared to those at baseline. The average change in DA metabolites was −4% from baseline, consistent with no effect of the drug. There were no significant correlations between the change in DA metabolite levels and the denervation severity.

## Discussion

The results presented in Figure 3 indicate that in the striatum ipsilateral to the lesioning, the EDVR of [^18^ F]FDOPA PET is reduced. This reduction was strongly correlated with the decrease in DA metabolite levels in the ECF as measured by microdialysis in 6-OHDA-lesioned rats. The observation of a reduction in DA metabolite concentrations in the ipsilateral striatum, which is well correlated with the degree of denervation (as assessed in our case via DTBZ binding, i.e., VMAT2 density) is in good agreement with the literature [32]. The data reported here are the first (to our knowledge) that directly compare such changes in the ECF with the results from recently developed [^18^ F]FDOPA PET imaging in rats; the comparison provides further verification of the imaging method since the findings for the ipsilateral striatum are in agreement with our predictions, considering the known effects of the lesioning. The method could provide further insight into the complexities of the dopaminergic system, somewhat paralleling the work of Hume et al. [33] who used [^11^C]raclopride (a dopamine D2/D3 receptor antagonist) PET imaging together with microdialysis in a similar rat model to study DA receptor supersensitivity after lesioning and the effects of acute l-DOPA administration. The work of Tsukada et al. [34] also makes use of similar techniques, using microdialysis to sample and analyze the various metabolites that occur in the rat brain after injection of the PET radiotracer l-[^11^C]-DOPA. The ability to distinguish between different radioactively labeled compounds in the ECF of the brain can be utilized to measure the metabolic and kinetic profiles of new radiotracers for PET imaging and to examine the effects of drugs on such profiles. In this study, we found entacapone to have little or no impact on DA and DA metabolite measures, supporting the view that kinetic parameters obtained from [^18^ F]FDOPA PET images are not altered by the use of this drug (which is required to prevent rapid peripheral metabolism of the radiotracer by COMT). Microdialysis is hence shown to complement PET in several studies that investigate the dopaminergic system.

The data also indicate that functional changes occur in the striatum contralateral to the 6-OHDA lesion, which are not accompanied by a large change in VMAT2 density nor in the steady-state vesicular storage of DA. In the striatum contralateral to the lesion, there were no reductions in [^11^C]DTBZ binding or in the EDVR of [^18^ F]FDOPA, but there was a significant difference (42%) in DA metabolite levels between the severely lesioned (>80%) rats and the mildly lesioned (<80%) rats. The origin of this difference is unclear since neither group's DA metabolite level was significantly different from that of the control rats. This lack of significance may be due to the low number of control rats and the variability in the measurements. It is possible that compensatory mechanisms lead to a small increase in DA metabolite levels in the contralateral striatum in mildly lesioned rats, and it appears likely that other mechanisms lead to reductions in DA metabolite levels in severely lesioned rats.

The suggestion of a reduction in DA metabolites in the ECF in the contralateral striatum seen here is similar to the trend observed in a previous work [35], but its exact cause remains unknown. The location of our 6-OHDA lesioning, the rostral end of the SNc, is caudal to the decussation of the nigrostriatal projection [36] and was hence expected to cause some damage to the crossed projection which originates in the ipsilateral SNc. Since crossed projections only contribute a few percent of the total striatal innervations, measures of VMAT2 density and DA storage in the contralateral striatum are not expected to be sensitive to the destruction or impairment of crossed projections, consistent with our observations of normal or near-normal VMAT2 density and DA storage in the contralateral striatum several months after lesioning. A reduction in DA metabolites in the ECF contralateral to the lesioning could be explained if the loss of crossed projections results in a disproportionately large reduction in neuronal activity or if the lesioning caused changes in other neuronal populations (e.g., substantia nigra reticular (SNr), pedunculopontine nucleus (PPN), or subthalamic nucleus (STN)) ipsilateral to the lesion which result in bilateral functional changes [37,38]. For extensive reviews of the 6-OHDA model, see [22,39]. Note that although DA concentrations were measured by microdialysis, our analysis purposefully focused mainly on the DA metabolite levels since these measures have proven to be much more consistent across the literature and they are expected to be more robust due to their far higher concentrations. That we did not observe a significant decrease in DA content in the ECF, nor any asymmetry between ipsilateral and contralateral striata, is in agreement with results from previous studies using 6-OHDA-lesioned rats with lesions of <95% and likely a result of compensatory mechanisms. In this respect, the DA concentration in ECF differs greatly from the total DA in tissue [32], which is a common measure of denervation severity and known to be very well correlated with both DTBZ binding (used here for measuring denervation severity) and the number of surviving DA terminals [11,12,29].

The increase in DA metabolites observed following benserazide/LDOPA in the striatum contralateral to lesioning was not dependent upon the degree of lesioning. This implies that compensatory changes that may occur in the contralateral striatum do not include a relative upregulation in the LDOPA response, despite the aforementioned decrease in the basal DA metabolite concentration for the striatum contralateral to lesioning. On the other hand, in the ipsilateral striatum, the more severely lesioned rats had an exaggerated response to LDOPA when considering the fractional increase in DA metabolite levels above the basal levels. This suggests that DA release is more dependent on the availability of exogenous levodopa in the ipsilateral (lesioned) striatum as compared to the contralateral striatum and to normal controls. The functional consequences of 6-OHDA lesioning thus appear to be different when considering the ipsilateral and contralateral striata, which strongly indicates that the changes in the contralateral striata are caused by different mechanisms to those changes in the striatum ipsilateral to the lesion, as may be expected [22]. Our data are consistent with compensatory effects that change basal DA metabolite levels in the contralateral striatum without a relative upregulation in the LDOPA response in the contralateral striatum and without an observable impact on the binding of DTBZ or on the EDVR and *k*_ref_ of [^18^ F]FDOPA for this contralateral region. In the ipsilateral striatum, all of these measures were reduced (although reductions in *k*_ref_ were attenuated [10]).

The absolute LDOPA-induced increase in DA metabolites in ECF was expected to be strongly correlated to the FDOPA PET-measured *k*_ref_. Recall that *k*_ref_ represents the rate-limiting steps for the conversion of exogenous LDOPA into vesicular-stored DA. A reduction in *k*_ref_ implies a reduction in the proportion of exogenous LDOPA converted into DA within the corresponding brain region, as compared to the proportion that is metabolized in the periphery. It is hence reassuring that measures of *k*_ref_ and the absolute LDOPA-induced increase in DA metabolites were strongly correlated. Basal DA metabolite levels appeared to be most strongly correlated with the degree of terminal loss in the ipsilateral striatum which is well quantified by the DTBZ BP_ND_, which is linearly related to the total DA storage capacity as estimated via the FDOPA EDVR in 6-OHDA-lesioned rats [10].

The reported DA and DA metabolite concentrations are those found after correction for probe recovery, using the *in vitro* measured recovery as an estimate of the *in vivo* recovery. It is known that these recoveries differ, and the values reported are hence not highly accurate measurements of levels in the ECF but are expected to be proportional to those values. The use of two different perfusion rates was accounted for by application of different probe recoveries, and to ensure that this procedure did not confound the results, we added probe perfusion rate as a covariate to a linear model (analysis of covariance (ANCOVA)); the rate was found to have no significant effects on the estimated DA metabolite levels.

The presented data provide a crucial link between two important investigative techniques: microdialysis (with analysis of DA and DA metabolites) and PET imaging (using [^18^ F]FDOPA and [^11^C]DTBZ). The investigation provides a means of relating data from these two techniques, but careful attention should be paid to the potential model-specific nature of the observed correlations: the reported findings are from the unilateral 6-OHDA rat model, with measurements made several months after an acute lesion. The effects of lesioning are expected to stabilize within 1 month and to far exceed age-related changes that could take place between the time of PET imaging and microdialysis. This view is strongly supported by the data, for example, the correlation found when assessing asymmetry (Figure 4), and also by our previous analyses of the effects of aging in this model [40]. We expect the observed relationships between the results from the various techniques to be applicable to some other models, but different relationships might be expected in models that differ greatly from that used here. Our comparison enables informed predictions of, for example, microdialysis-measured DA metabolite concentrations, using as an input the data collected at various time points throughout a longitudinal PET study. Different investigations may hence be compared in this way to assess their agreement. In addition to this, the comparison aids in the interpretation of results from [^18^ F]FDOPA PET imaging, providing reassurances that the biological interpretations assigned to the various parameter estimates (i.e., EDVR and *k*_ref_) are valid. The presented data build on our previous work [10], which examined the effects of central COMT inhibition on [^18^ F]FDOPA PET parameter estimates, and made a comparison between [^18^ F]FDOPA and [^11^C]DTBZ PET measures in these lesioned rats. Also crucial to the verification of [^18^ F]FDOPA PET methodology for rodent imaging was the work of Kyono et al. [9] who compared the results from [^18^ F]FDOPA PET in unilaterally 6-OHDA-lesioned rats (lesioned at the striatum rather than at the SNc as here) with assays of brain homogenates (measurement of DA and DA metabolites in striatal tissue) and brain sections (tyrosine hydroxylase immunohistochemistry) and with results from behavioral testing. Results from imaging closely matched those from immunohistochemistry, with tracer uptake also correlating strongly with DA (and DA metabolite) concentrations in tissue and with turning behavior. These previous works demonstrated the utility of [^18^ F]FDOPA PET imaging in rats and verified that such imaging was sensitive to the effects of 6-OHDA lesioning and pharmacological reduction in DA turnover. Using *in vivo* microdialysis, we furthered these works by measuring the DA and DA metabolite content in the ECF (as opposed to the whole tissue) at baseline and following the administration of LDOPA, providing greater verification of the imaging method and providing new insights into the changes that can take place in the striatum contralateral to lesioning in the 6-OHDA model.

## Conclusions

To conclude, microdialysis and PET ([^18^ F]FDOPA and [^11^C]DTBZ) are sensitive techniques for assessment of the functionality of the dopaminergic system and may both be crucial in furthering our understanding of diseases such as Parkinson's. These techniques are vastly different (invasive direct sampling vs. non-invasive neuroimaging) and provide distinct information regarding the dopaminergic system. In some cases, as found here in the striatum ipsilateral to 6-OHDA lesioning in the rat, the results may be highly correlated. In other cases, such as for the striatum contralateral to the lesioning in this model, these metrics of dopaminergic function may diverge. Such observations can provide insights into specific functional changes occurring at the molecular level, highlighting the synergy of multimodel investigations.

## Abbreviations

6-OHDA: 6-hydroxydopamine; AADC: Amino acid decarboxylase; BPND: Binding potential; COMT: Catechol-*O*-methyltransferase; DA: Dopamine; DOPAC: 3,4-dihydroxyphenylacetic acid; DTBZ: Dihydrotetrabenazine; ECF: Extracellular fluid; EDVR: Effective distribution volume ratio; FDOPA: Fluoro-3,4-dihydroxyphenyl-l-alanine; HVA: Homovanillic acid; LDOPA: Levodopa; OMFD: 3-*O*-methyl-6-fluoro-l-dopa; PD: Parkinson's disease; PET: Positron emission tomography; SNc: Substantia nigra pars compacta; VMAT2: Vesicular monoamine transporter 2.

## Competing interests

The authors declare that they have no competing interests.

## Authors’ contributions

MW was involved in the study design and the acquisition, analysis, and interpretation of all data, and wrote the manuscript. KD helped design and run the study. RK advised on the study design, carried out the surgeries, and was involved in all data acquisition. AL was involved in the acquisition and analysis of microdialysis data. MJF, AJS, and VS were involved in the study design and interpretation of results. All authors read and improved the final manuscript.

## References

[B1] Garnett ES Firnau G Nahmias C Dopamine visualized in the basal ganglia of living man Nature 1983 3 137 138 10.1038/305137a0 6604227

[B2] Gjedde A Reith J Dyve S Leger G Guttman M Diksic M Evans A Kuwabara H Dopa decarboxylase activity of the living human brain Proc Natl Acad Sci USA 1991 3 2721 2725 10.1073/pnas.88.7.2721 1688340 PMC51310

[B3] Kumakura Y Cumming P PET studies of cerebral levodopa metabolism: a review of clinical findings and modeling approaches Neuroscientist 2009 3 635 650 10.1177/1073858409338217 19793723

[B4] Martin WRW Palmer MR Patlak CS Calne DB Nigrostriatal function in humans studied with positron emission tomography Ann Neurol 1989 3 535 542 10.1002/ana.410260407 2510586

[B5] Holden JE Doudet D Endres CJ Chan GLY Morrison KS Vingerhoets FJG Snow BJ Pate BD Sossi V Buckley KR Ruth TJ Graphical analysis of 6-fluoro-L-dopa trapping: effect of inhibition of catechol-O-methyltransferase J Nucl Med 1997 3 1568 1574 9379194

[B6] Sossi V Doudet DJ Holden JE A reversible tracer analysis approach to the study of effective dopamine turnover J Cereb Blood Flow Metab 2001 3 469 476 11323532 10.1097/00004647-200104000-00015

[B7] Sharma SK Ebadi M Distribution kinetics of F-18-DOPA in weaver mutant mice Mol Brain Res 2005 3 23 30 10.1016/j.molbrainres.2005.05.018 15979197

[B8] Sharma SK El-ReFaey H Ebadi M Complex-1 activity and F-18-DOPA uptake in genetically engineered mouse model of Parkinson's disease and the neuroprotective role of coenzyme Q(10) Brain Res Bull 2006 3 22 32 10.1016/j.brainresbull.2005.11.019 16750479

[B9] Kyono K Takashima T Katayama Y Kawasaki T Zochi R Gouda M Kuwahara Y Takahashi K Wada Y Onoe H Watanabe Y Use of [18F]FDOPA-PET for in vivo evaluation of dopaminergic dysfunction in unilaterally 6-OHDA-lesioned rats EJNMMI Res 2011 3 25 10.1186/2191-219X-1-25 22214344 PMC3251329

[B10] Walker MD Dinelle K Kornelsen R McCormick S Mah C Holden JE Farrer MJ Stoessl AJ Sossi V In-vivo measurement of LDOPA uptake, dopamine reserve and turnover in the rat brain using [18F]FDOPA PET J Cereb Blood Flow Metab 2013 3 59 66 10.1038/jcbfm.2012.120 22929441 PMC3597374

[B11] Masuo Y Pelaprat D Scherman D Rostene W [H-3]Dihydrotetrabenazine, a new marker for the visualization of dopaminergic denervation in the rat striatum Neurosci Lett 1990 3 45 50 10.1016/0304-3940(90)90426-A 1974339

[B12] Sossi V Holden JE Topping GJ Camborde ML Kornelsen RA McCormick SE Greene J Studenov AR Ruth TJ Doudet DJ In vivo measurement of density and affinity of the monoamine vesicular transporter in a unilateral 6-hydroxydopamine rat model of PD J Cereb Blood Flow Metab 2007 3 1407 1415 10.1038/sj.jcbfm.9600446 17245418

[B13] Benveniste H Brain microdialysis J Neurochem 1989 3 1667 1679 10.1111/j.1471-4159.1989.tb07243.x 2656913

[B14] Darvesh AS Carroll RT Geldenhuys WJ Gudelsky GA Klein J Meshul CK Van der-Schyf CJ In vivo brain microdialysis: advances in neuropsychopharmacology and drug discovery Expert Opin Drug Discov 2011 3 109 127 10.1517/17460441.2011.547189 21532928 PMC3083031

[B15] Ungerstedt U Microdialysis—principles and applications for studies in animals and man J Intern Med 1991 3 365 373 10.1111/j.1365-2796.1991.tb00459.x 1919432

[B16] Del-Amo EM Urtti A Yliperttula M Pharmacokinetic role of L-type amino acid transporters LAT1 and LAT2 Eur J Pharm Sci 2008 3 161 174 10.1016/j.ejps.2008.06.015 18656534

[B17] Deep P Gjedde A Cumming P On the accuracy of an [18F]FDOPA compartmental model: evidence for vesicular storage of [18F]fluorodopamine in vivo J Neurosci Methods 1997 3 157 165 10.1016/S0165-0270(97)00094-0 9350967

[B18] Peter D Liu Y Sternini C De-Giorgio R Brecha N Edwards RH Differential expression of two vesicular monoamine transporters J Neurosci 1995 3 6179 6188 7666200 10.1523/JNEUROSCI.15-09-06179.1995 PMC6577657

[B19] Okada M Nakao R Hosoi R Zhang MR Fukumura T Suzuki K Inoue O Microdialysis with radiometric monitoring of L-[beta-(11)C]DOPA to assess dopaminergic metabolism: effect of inhibitors of L-amino acid decarboxylase, monoamine oxidase, and catechol-O-methyltransferase on rat striatal dialysate J Cereb Blood Flow Metab 2011 3 124 131 10.1038/jcbfm.2010.58 20407462 PMC3049477

[B20] Westerink BH Sequence and significance of dopamine metabolism in the rat brain Neurochem Int 1985 3 221 227 10.1016/0197-0186(85)90108-1 20492917

[B21] Ungerstedt U 6-Hydroxy-dopamine induced degeneration of central monoamine neurons Eur J Pharmacol 1968 3 107 110 10.1016/0014-2999(68)90164-7 5718510

[B22] Schwarting RKW Huston JP Unilateral 6-hydroxydopamine lesions of meso-striatal dopamine neurons and their physiological sequelae Prog Neurobiol 1996 3 215 266 10.1016/S0301-0082(96)00015-9 8878304

[B23] Paxinos G Watson C The Rat Brain in Stereotaxic Coordinates 1997 San Diego: Academic 10.1016/0165-0270(80)90021-7 6110810

[B24] Kelly PH Iversen SD Selective 6OHDA-induced destruction of mesolimbic dopamine neurons: abolition of psychostimulant-induced locomotor-activity in rats Eur J Pharmacol 1976 3 45 56 10.1016/0014-2999(76)90352-6 1033072

[B25] Kim JS Lee JS Im KC Kim SJ Kim SY Lee DS Moon DH Performance measurement of the microPET focus 120 scanner J Nucl Med 2007 3 1527 1535 10.2967/jnumed.107.040550 17704248

[B26] McLellan CA Doudet DJ Brucke T Aigner TG Cohen RM New rapid analysis method demonstrates differences in 6-(F-18)fluoro-L-dopa plasma input curves with and without carbidopa and in hemi-MPTP lesioned monkeys Appl Radiat Isot 1991 3 847 854 10.1016/0883-2889(91)90223-N 1657833

[B27] Rubins DJ Melega WP Lacan G Way B Plenevaux A Luxen A Cherry SR Development and evaluation of an automated atlas-based image analysis method for microPET studies of the rat brain Neuroimage 2003 3 2100 2118 10.1016/j.neuroimage.2003.07.011 14683714

[B28] Logan J Fowler JS Volkow ND Wang GJ Ding YS Alexoff DL Distribution volume ratios without blood sampling from graphical analysis of PET data J Cereb Blood Flow Metab 1996 3 834 840 8784228 10.1097/00004647-199609000-00008

[B29] Topping GJ Dinelle K Kornelsen R McCormick S Holden JE Sossi V Positron emission tomography kinetic modeling algorithms for small animal dopaminergic system imaging Synapse 2010 3 200 208 10.1002/syn.20716 19862685

[B30] Patlak CS Blasberg RG Graphical evaluation of blood-to-brain transfer constants from multiple-time uptake data. Generalizations J Cereb Blood Flow Metab 1985 3 584 590 10.1038/jcbfm.1985.87 4055928

[B31] Levin JR Serlin RC Seaman MA A controlled, powerful multiple-comparison strategy for several situations Psychol Bull 1994 3 153 159

[B32] Robinson TE Whishaw IQ Normalization of extracellular dopamine in striatum following recovery from a partial unilateral 6-OHDA lesion of the substantia nigra: a microdialysis study in freely moving rats Brain Res 1988 3 209 224 10.1016/0006-8993(88)91560-0 3135914

[B33] Hume SP Opacka-Juffry J Myers R Ahier RG Ashworth S Brooks DJ Lammertsma AA Effect of L-dopa and 6-hydroxydopamine lesioning on [11C]raclopride binding in rat striatum, quantified using PET Synapse 1995 3 45 53 10.1002/syn.890210107 8525461

[B34] Tsukada H Lindner KJ Hartvig P Tani Y Bjurling P Kihlberg T Westerberg G Watanabe Y Langstrom B Effect of 6R-L-erythro-5,6,7,8-tetrahydrobiopterin on in vivo L-[beta-11C]dopa turnover in the rat striatum with infusion of L-tyrosine J Neural Transm Gen Sect 1994 3 1 15 10.1007/BF01283026 7857582

[B35] Curran EJ Albin RL Becker JB Adrenal medulla grafts in the hemiparkinsonian rat: profile of behavioral recovery predicts restoration of the symmetry between the two striata in measures of pre- and postsynaptic dopamine function J Neurosci 1993 3 3864 3877 8103552 10.1523/JNEUROSCI.13-09-03864.1993 PMC6576439

[B36] Douglas R Kellaway L Mintz M Van-Wageningen G The crossed nigrostriatal projection decussates in the ventral tegmental decussation Brain Res 1987 3 111 121 10.1016/0006-8993(87)90967-X 3117325

[B37] Gonzalez-Hernandez T Barroso-Chinea P Rodriguez M Response of the GABAergic and dopaminergic mesostriatal projections to the lesion of the contralateral dopaminergic mesostriatal pathway in the rat Mov Disord 2004 3 1029 1042 10.1002/mds.20206 15372592

[B38] Breit S Martin A Lessmann L Cerkez D Gasser T Schulz JB Bilateral changes in neuronal activity of the basal ganglia in the unilateral 6-hydroxydopamine rat model J Neurosci Res 2008 3 1388 1396 10.1002/jnr.21588 18061958

[B39] Schwarting RK Huston JP The unilateral 6-hydroxydopamine lesion model in behavioral brain research. Analysis of functional deficits, recovery and treatments Prog Neurobiol 1996 3 275 331 10.1016/S0301-0082(96)00040-8 8971983

[B40] Dinelle K Walker MD McCormick S Kornelsen R Sossi V Age related changes in the dopaminergic system of the Sprague–Dawley rat measured via positron emission tomography [abstract] J Nucl Med 2013 3 Suppl 2 249

